# Comparative study of the metal accumulation in *Hysterothalycium reliquens* (nematode) and *Paraphilometroides nemipteri* (nematode) as compared with their doubly infected host, *Nemipterus peronii* (Notched threadfin bream)

**DOI:** 10.1007/s00436-014-4039-x

**Published:** 2014-08-14

**Authors:** Roshan Mazhar, Noor Azhar Shazili, Faizah Shaharom Harrison

**Affiliations:** Institute of Tropical Aquaculture, Universiti Malaysia Terengganu, 21030 Kuala Terengganu, Malaysia

**Keywords:** Biomonitoring, Metal pollution, Accumulation, South China Sea

## Abstract

In February 2013, forty-seven Notched threadfin bream, the *Nemipterus peronii*, were sampled from the eastern coastal waters of the South China Sea. The concentration of various elements, namely cadmium (Cd), chromium (Cr), copper (Cu), mercury (Hg), strontium (Sr), manganese (Mn), selenium (Se), Lead (Pb), nickel (Ni), aluminum (Al), arsenic (As), iron (Fe), and Zinc (Zn) were analyzed in the liver, muscle, and kidney organs of the host, as well as in their parasites *Hysterothalycium reliquens* (nematode) and the *Paraphilometroides nemipteri* (nematode), using inductively coupled plasma mass spectrometry (ICP-MS). The former group of parasites showed highest accumulation capacity for Cr, Cu, Fe, Mn, Se, Ni, and Zn while the latter group had high accumulation potential of As, Hg, Cd, Al, Pb, and Sr. The divergence in heavy-metal accumulation profiles of both nematodes is linked with the specificity of microhabitats, cuticle morphology, and interspecific competition. The outcome of this study indicates that both parasite models can be used for biomonitoring of metal pollution in marine ecosystems.

## Introduction

“Is it worth pursuing the use of parasites as indicators of marine pollution?” (Mackenzie 2008). Recently, wide applications of parasitological models, used to investigate heavy metals pollution in different ecosystems, affirm the answer to this pertinent question. Data on parasite-host systems, as bio indicators of heavy metal pollution in the marine environment, is still scarce considering that the available information mainly concerns freshwater or estuarine ecosystems. Presently, the available data on parasites of marine hosts refer to only a few cestode and nematode species (Mendes et al. 2013). In fact, nematodes as bioaccumulators had received very inconsistent attention unlike other groups of helminthes; over the last two decades, acanthocephalan has been coherently studied, well documented and established as the perfect sentinel (Sures et al. 1994a, b; 1997a, b, c; 1998; 1999, 1999a, b, 2003; Sures and Taraschewski 1994, 1995; Sures 2003a, b; Sures and Reimann 2003; Sures and Siddall 2001, 2003).

Metal monitoring studies performed with the help of parasitic nematodes are comparatively scarce (Nachev et al. 2013), though nematodes accord to the criteria summarized by (Sures 2003a) for selecting sentinels in environmental monitoring, in terms of abundance, accumulation capacities, excellent environmental reflecting, and their adaptive flexibilities against unfavorable conditions.

Anisakids nematodes characteristically occur in deep waters as meso- or benthopelagic species and are typically found in predators (Abollo et al. 2001). They have distinct advantages over other types of biomarkers; they have a high pollution tolerance, and from both the logistical and economical points of view relatively sensitive markers. They also occupy all the trophic levels of the food web in all aquatic ecosystems. They are readily available at low cost during routine fishing programs and standard necropsy procedures. Ease of technique (i.e., ease of collection and identification) is also a key factor that enhances the suitability of this parasite tag (Pascual and Abollo 2005).

There have been few comparative studies where intestinal parasites may be used as sentinel organisms to monitor the concentration of bioavailable metals, not only in fresh water but also in estuarine and marine ecosystems. The most abundant fish anisakid nematode species, of the genus *Hysterothylacium*, are an extremely common digestive tract parasite of teleosts, especially in the marine environnent (Navone et al. 1998). Anisakid nematodes of the genus *Hysterothylacium* use fish as both intermediate and definitive hosts in which they attain maturity. Physiological studies (Pascual and Abollo 2003) concerning the accumulation of heavy metals in adults and larvae of Anisakis have reported them as efficient accumulators. Khaleghzadeh-ahangari et al. (2011) also confirmed the importance of *Hysterothalycium* MB larvae as bioindicators of heavy metals, and their potential use in environmental studies.

Philometrids are primarily histozoic nematodes that are exclusively fish parasites as adults. Although fairly common and potentially pathogenic to their hosts, current knowledge of this group is quite limited (Moravec et al. 2004, 2006). Among the 28 species of the genus *Philometra* that parasitize fish, there were two nematode parasites (*Philometra ovata and Philometra cyprinirutili* from philometroid group) of freshwater cyprinids that possess the necessary characteristics to act as sentinel organisms (Moravec et al. 2004). While there may be other parasite species that could be suitable for use as indicators of heavy metals’ load in aquatic ecosystems (Barus et al. 2007), this group displayed the most variation in their ability to bioaccumulate heavy metals with adult philometrids (Tenora et al. 2000; Barus et al. 2007), whereas other species displayed little or no concentration of some, not all metals (Szefer et al. 1998; Tenora et al. 1999; Barus et al. 2001; Palikova and Barus 2003).

The use of bioindicators to study the heavy metals pollution in the Malaysian environment has received widespread attention; shellfish, which includes bivalves, gastropods, and crustaceans, have been used in bioaccumulation metals studies (Franklin and Edward 2009; Mokhtar 2009). Fish parasites, as models in studies of heavy metals and pollutant accumulations, have never been taken into account for biomonitoring in the South China Sea. As such, this study is the first attempt, wherein two different nematodes are used as parasitological models, to collate their bioaccumulation capacities with that of host tissues inhabiting mid-continental shelf region of eastern coastal waters of the South China Sea.

## Material and methods

### Fish tissues and parasites collection

**Fig. 1:**
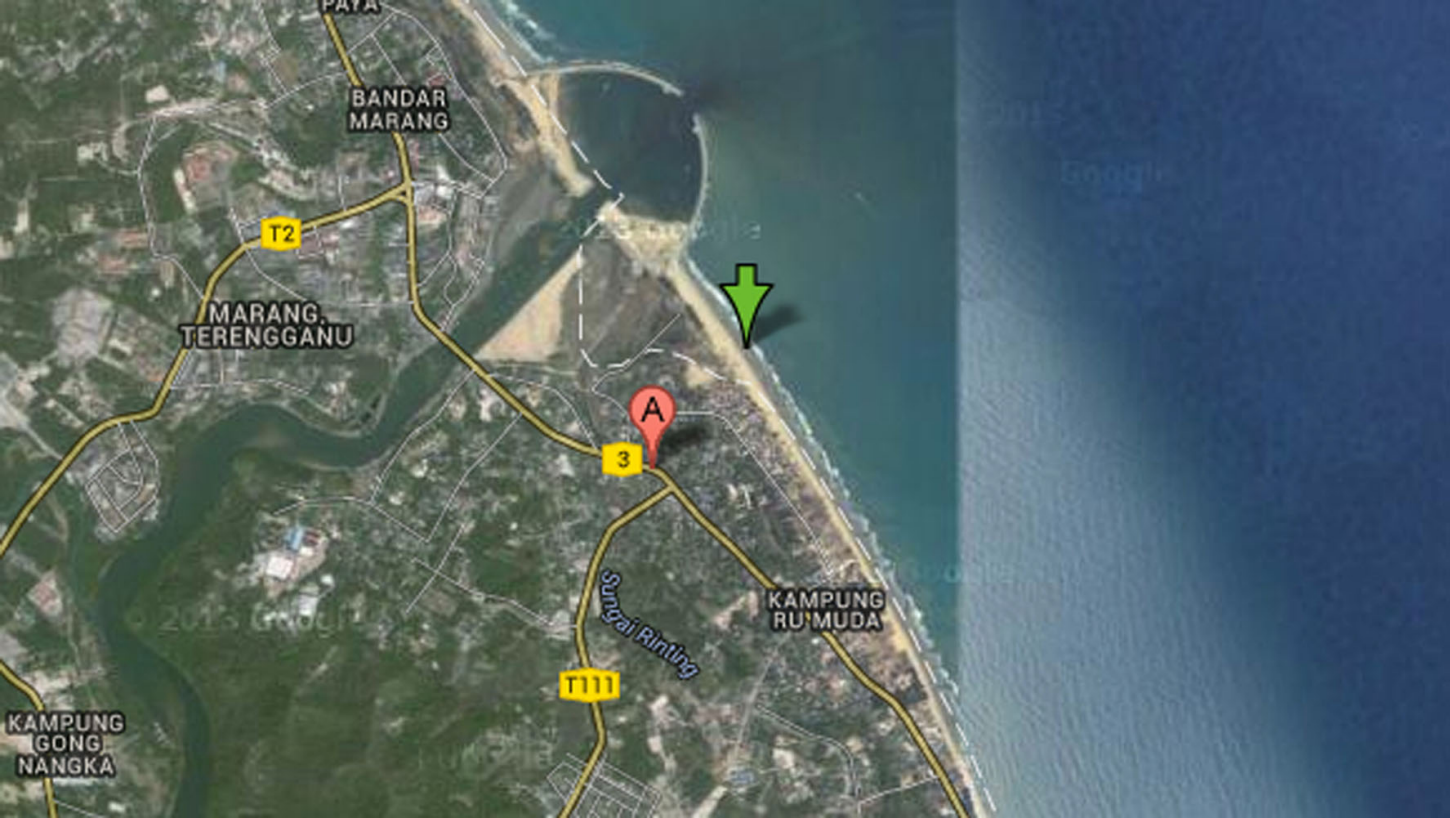
Location of sampling site

A total of forty-seven Notched threadfin bream (*Nemipterus peronii*) were collected via trawl net from Marang, a mid-shelf region off the eastern coast of the South China Sea coast (5°12′ N, 103°13′ E), also known as a jigging spot of squids where notched threadfin bream are the most prevailing fish species. The sampling location is shown in the Fig. 1.

It should be noted that in this area a heavy influx of tourist and other fleet movements exert huge anthropogenic pressure. The collected samples were brought to the laboratory on the same day. Prior to dissection, the total length and weight of all individuals were recorded. After dissection each fish’s fins, operculum and body scales were examined, and parasites were collected using stainless steel instruments and MiliQ water. The whole intestinal tract was removed from each fish, the worms were carefully removed, using the aforementioned instruments, and placed in pre-cleaned bottles with lids. Thereafter the worms were stored at − 20 °C until further processing.

Samples of the muscles, liver, and kidneys of all individuals were also taken and placed in clean plastic vials with lids. They were also kept frozen at −20 °C until metal analysis was done. Each sample tissues with 0.05 g (dry weight) as well as of the parasites was accurately weighed and placed in a Teflon vessel. Then, 2 ml HNO_3_ (65 % Suprapur Merck, D-6100 Darmstadt, Germany) and 1 ml H_2_O_2_ (30 % Merck, D-6100 Schuehardt, Germany) were added to the Teflon vessel, which was heated at 100 °C for 5 h and left overnight.

After completion of digestion process the samples were diluted with deionized water to a volume of 10 ml, and then analyzed for trace elements in an inductively coupled plasma mass spectrometer (ICP-MS-Perkin Elmer ELAN6100) at the Institute of Oceanography. Concentrations of Cd, Mn, Zn, Cu, Se, As, Sr, Al, Ni, Fe, Cr, Hg, and Pb were recorded (Table 2).

### Analytical procedure

In order to determine the accuracy of the extraction procedure, a standard reference material (SRM: DORM-3, NRCC) was applied using the same protocol (Table 1). The recovery range for heavy metals must be between 90 % and 100 %.

**Table 1:** Trace metal concentrations in certified reference material (DORM-3) as well as accuracy and determined by ICP-MS analyses

Metals	Certified value	ICPMS value	Accuracy (%)
Cr	1.89 ± 0.17	1.82 ± 0.32	93
Ni	1.28 ± 0.24	1.15 ± 0.81	90
Cd	0.29 ± 0.02	0.28 ± 0.05	96
As	6.8 ± 0.30	6.32 ± 0.23	92
Cu	15.5 ± 0.63	14.1 ± 0.33	91
Pb	0.39 ± 0.05	0.38 ± 0.02	97
Hg	0.38 ± 0.06	0.35 ± 0.14	92
Zn	51.3 ± 3.1	48.4 ± 6.7	95
Fe	347 ± 20	315 ± 43	91

### Statistical analysis

The Graph Pad PRISM 5.0 statistical package was used for statistical analysis. The one-way ANOVA method was applied followed by nonparametric Tukey’s multiple comparison test method. The bioconcentration factors (BCF) were computed according to Sures et al. (1999a), as the ratios *C*(parasite)/*C*(host tissue).The bioaccumulation ratio between both nematodes, *Paraphilometroides nemipteri and Hysterothalycium reliquens*, was also calculated. Spearman rank correlation was performed to find all possible relationships among the element concentrations in different organs of host and both nematodes. Table 2 illustrates the calculated results from the survey.

**Table 2:** Metal concentration (microgram per gram dry weight) in tissues of *Nemipterus peroni* and its parasites *Hysterothalycium reliquens* and *Paraphilometroides nemipteri (*mean values ± SD)

Metals	*H .reliquens*	*P. nemipteri*	Muscle	Liver	Kidney
Cr	4.26 ± 0.16	1.22 ± 0.35	0.31 ± 0.07	0.33 ± 0.0	0.39 ± 0.04
Fe	51.8 ± 2.7	48.8 ± 2.7	1.9 ± 0.54	19.2 ± 6.9	31.3 ± 1.7
Zn	17.3 ± 3.3	13.0 ± 2.5	0.52 ± 0.10	2.26 ± 0.72	2.18 ± 1.7
Cd	0.07 ± 0.08	0.117± 0.02	0.0004 ± 0.0	0.03 ± 0.03	0.03 ± 0.003
Ni	1.21 ± 0.19	0.55 ± 0.51	0.05 ± 0.02	0.11 ± 0.03	0.13 ± 0.04
Cu	1.51 ± 1.21	1.3 ± 0.40	0.025 ± 0.01	0.31 ± 0.11	0.36 ± 0.11
Hg	0.05 ± 0.02	0.06 ± 0.00	.004	0.064 ± 0.023	0.02 ± 0.00
Sr	4.95 ± 2.00	9.0 ± 5.94	0.036 ± 0.007	0.15 ± 0.02	0.085 ± 0.03
Al	4.0 ± 2.14	14.7 ± 3.3	0.521 ± 0.2	0.23 ± 0.11	0.37 ± 0.03
As	3.24 ± 0.1	4.71 ± 0.16	0.13 ± 0.04	2.52 ± 0.07	1.52 ± 0.22
Pb	0.26 ± 0.03	0.44 ± 0.03	0.04 ± 0.02	0.05 ± 012	0.12 ± 0.028
Mn	1.85 ± 0.58	1.70 ± 0.51	0.027 ± 0.003	0.19 ± 0.08	0.184 ± 0.04
Se	1.18 ± 1.64	0.39 ± 0.17	0.075 ± 0.04	0.25 ± 0.1	0.54 ± 0.21

## Results

**Fig. 2:**
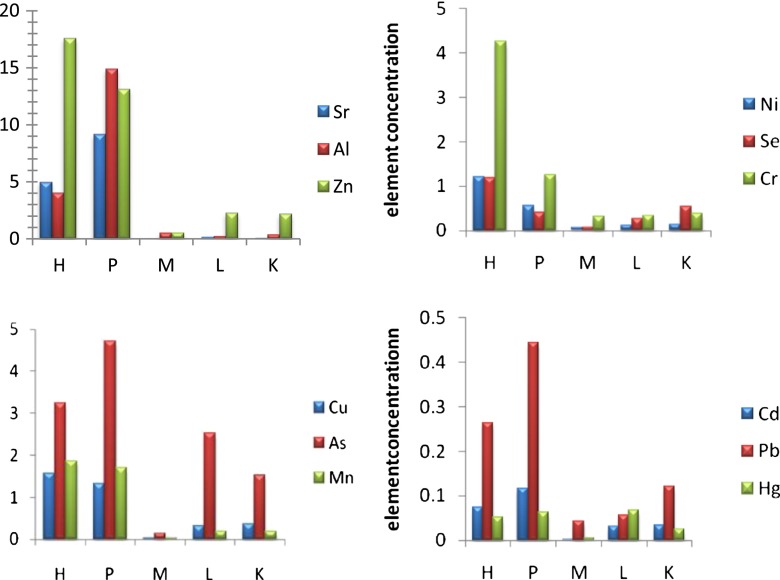
Comparison of mean concentrations (dry weight, ug/g) of elements in selected organs of *Nemipterusperoni* and in its parasites. *H Hysterothalycium.reliquens*, *P Paraphilometroides nemipteri*, *M* muscle, *L* liver, and *K* kidney

The adult *P .nemipteri* was detected in 31 Notched threadfin bream, showing a prevalence of 69 % and an intensity of eight (3–14) parasites. *H. reliquens* was detected in 43 Notched threadfin bream, indicating a prevalence of 97 % with an intensity of 14 (7–32). The following results refer to a group of 29 Notched threadfin bream, all doubly infected with *P. nemipteri,* as well as with *H. reliquens.* Figure 2 shows the selected elements concentrations in both the nematodes and the host organs.

### Elements concentrations in both nematodes and host organs

The upshot of analyzed data illustrated that both parasites’ nematodes, *H. reliquens* and *P. nemipteri* had significantly higher concentration potentials for all 13 elements than their common host fish tissues (Fig. 2). The concentration profile of *H. reliquens*, in comparison to the host tissues, was identified with significantly higher potential for all essential elements (except Cr) Mn, Zn, Fe Ni, Se, and Cu and its counterpart *P. nemipteri* which were in descending order as Mn > Cu > Zn > Fe > Ni > Se > Cr (Tukey’s test), as detailed in Table 2.

**Table 3:** Bioconcentration factors [*C*(parasite)/*C*(Notched thread bream tissue] for *H. reliquens* and *P. nemipteri*, calculated with respect to different host tissues and their ratios [*C*(*P. nemipteri*)/*C*(*H. reliquens)*]

Metals	*H. reliquens*			*P. nemipteri*			H/P
	Muscle	Liver	Kidney	Muscle	Liver	Kidney	
Cr	13.7 ± 2.1***	12.9 ± 1.9***	10.8 ± 2.1***	4.04 ± 0.16***	3.7 ± 0.51*	3.2 ± 0.4**	3.4 ± 1.6***
Fe	27.02 ± 9.8*	2.6 ± 0.76*	1.6 ± 2.9	25.4 ± 5.3	2.5 ± 0.41	1.5 ± 1.6*	1.0 ± 1.8**
Zn	33.3 ± 9***	7.7 ± 8***	7.95 ± 8***	24.2 ± 7***	5.7 ± 6**	5.9 ± 6**	1.3 ± 1.0*
Ni	24.2 ± 0.15**	10.7 ± 0.4**	8.6 ± 0.02**	11 ± 0.02	4.8 ± 0.03	3.9 ± 0.13	2.2 ± 0.5*
Cu	60.1 ± 5	4.7 ± 4	4.2 ± 7	52.3 ± 2	4. ± 1.1	3.5 ± 3	1.16 ± 2
Pb	6.7 ± 4.3**	5.36 ± 6.9**	2.21 ± 2.5*	11 ± 1.9***	8.80 ± 3.0***	3.6 ± 1.7***	1.6 ± 0.31**
Mn	68.1 ± 47**	9.5 ± 6**	10.0 ± 12**	62.7 ± 46**	8.7 ± 6*	9.2 ± 12*	1.08 ± 1
Sr	137.1 ± 3.7*	32 ± 2.9	57.7 ± 2	248 ± 6.2*	60 ± 6.1*	104 ± 6.3*	1.8 ± 4.5*
Cd	185 ± .06*	2.05 ± 0.06***	2.2 ± 0.06	292 ± 0.06*	3.25 ± 0.03***	3.5 ± 0.02*	1.5 ± 0.05*
Al	7.6 ± 2*	17.4 ± 2.1	10.6 ± 2.4	28 ± 8*	64 ± 8.4*	39 ± 8.1*	3.6 ± 6*
Se	15.7 ± 1.2***	4.5 ± 1.7	2.1 ± 1.3	5.2 ± 2	1.5 ± 0.11	ND	2.9 ± 1.1
Hg	12.75 ± 0.03	0.77 ± 0.028*	2.12 ± 0.022***	15.5 ± 0.033	0.93± 0.021	2.5 ± 0.021*	1.2 ± 0.01**
As	24 ± 1.73*	1.2 ± 0.40***	2.1 ± 0.95***	34 ± 2.6	1.861.2	3.09 ± 1.75*	1.4 ± 0.81*

*ND* not detected

Tukeys test method = *p < 0.05; **p < 0.01; ***p < 0.001

The highest bioaccumulation level of Mn showed in *H. reliquens* has BCF = 68 (*P* < 0.01), while the maximum bioaccumulation ratio of a nonessential element (when comparing to host muscles) was recorded for Cd at BCF = 185 (*P* < 0.05), followed by Sr. Spearman’s correlation analysis which revealed several significant positive associations between the element concentrations in all host tissues and *H. reliquens* parasites. Some strong, significant associations for Mn, Cu, Zn, Fe, Se, Pb, Cd, and Al are shown in the Table 4.

**Table 4:** Spearman correlation coefficients (*R*) for the significant relationships between element concentrations in parasites and fish tissues

Metals	Parasites 1 vs. host	*R*	*P*	Parasites 2 vs. host	*R*	*P*
Se	*H. reliquens ×* muscle *H. reliquens × .liver*	0.98 0.65	<0.001 <0.05	*P. nemipteri ×* muscle	0.68	<0.05
Cd	*H. reliquens ×* liver	0.98	<0.001	*P. nemipteri × H. reliquens* *P. nemipteri ×* kidney	0.63 0.66	<0.05 <0.05
As	*H. reliquens ×* liver *H. reliquens ×* kidney	0.90 0.98	<0.001 <0.001	*P. nemipteri × H. reliquens*	0.63	<0.05
Pb	*H. reliquens ×* kidney	0.78	<0.01	*P. nemipteri × muscle* *P. nemipteri × .liver*	0.82 0.96	<0.01 <0.001
Fe	*H. reliquens × P. nemipteri*	0.84	<0.001			
Sr	*H. reliquens ×* kidney *H. reliquens ×* liver	0.85 0.97	<0.001 <0.001	*P. nemipteri ×* muscle	0.85	<0.001
Al	*H. reliquens ×* kidney	0.83	<0.001	*P. nemipteri × H. reliquens*	0.62	<0.05
Mn	*H. reliquens ×* liver	0.59	<0.05	*P. nemipteri ×* muscle	0.94	<0.001
Zn	*H. reliquens ×* muscle	0.80	<0.001	*P. nemipteri × H.* reliquens	0.50	<0.05

*P. nemipteri* showed exclusive bioaccumulation profile of trace elements; all toxic elements, including Cd, Pb and Sr, As, Al, and (Table 2), possessed significantly higher concentration levels in *P. nemipteri* than in the remaining selected fish tissues and *H. reliquens.* The highest bioaccumulation factor found was for Cd, being 292 times higher in *P. nemipteri* than in fish muscles (BCF = 292; *P* < 0.05), followed by Sr, As, Al, Hg, and Pb.

Although the mean BCF values of Cr in *P. nemipteri* were lower than other toxic metals, it remained significantly high (BCF = 4.04, 3.7, 3.2; *P* < .001, *P* < .05, and *P* < .01) relative to the host muscle, liver, and kidney, respectively. Several strong positive relations were also detected between the selected elements’ concentrations in *P. nemipteri* and host tissues.

However, both parasitic nematodes *H. reliquens* and *P. nemipteri* have shown different selective profiles of metal bioaccumulation. All the essential elements except Cr (including Fe, Cu, Zn, Ni, Se, and Mn) were found in significantly higher concentration in *H. reliquens* (Table 3). In contrast, *P. nemipteri* exhibited higher concentrations of nonessential elements such as Cd, Pb, Al, As, Sr, and Hg. A number of strong significant positive correlations were detected for As, Cr, Cd, Al, Fe, Ni, and Zn for both parasitic nematodes. The highest bioaccumulation ratio of Al was recorded at (BCF = 3.6; *P* < 0.05) followed by the ratios of Cr, Se, Ni, Mn, Sr, Zn, Cu, and Fe, all found greater than 1, while the lowest BCF was calibrated for Fe valued as (BCF = 1.0; *P* < 0.01).

## Discussion

The present outcome exhibited disparity between the accumulation capacities of both parasitic nematodes and those of their common host tissues (for all selected metals), which might be linked to various factors such as specific features of the nematode’s cuticle, specification of microhabitats, and other complex causes.

*H. reliquens* showed a significantly high tendency to accumulate all essential elements except Cr, while *P. nemipteri* indicated high affiliation for nonessential elements: the concentration of the six nonessential elements Pb, Cd, As, Sr, Hg, and Al were recorded significantly higher in *P. nemipteri* (relative to its counterpart as well as host tissues). For instance, accumulation for Cd was 292, Sr was 248, and Pb was 11 times higher than in host muscle tissues. On the other hand, *H. reliquens* displayed a higher accumulation level of elements such as Fe, Cu, Zn, Ni, and Mn.

The present findings strongly support the hypothesis that the cuticle’s role seems to be important in absorbing substances from the environment (Szefer et al. 1998); both nematodes showed a wide morphological variation of cuticle structure. *P. nemipteri* possesses a highly porous, thin cuticle that the internal organs can be seen through, ornamented with numerous, transversely elongated excrescences (Moravec 2010), which enhanced the surface area for the absorption, contrastingly, an adult *H. reliquens* has thick integument due to continuous molting (Cox et al. 1981b), which may hamper bile absorption in high concentration (Zehra et al. 2013). Compared to previous findings, the results showed that the bioaccumulation ratio of Cd in *P. nemipteri* was 268 times higher than host muscle, while ratios of Pb and Cr were 9,117 times lower than *P. ovata. P. nemipteri* also revealed high accumulation potential for Zn and Cu, being 21 and 30 times higher than in *P. ovata*, respectively.

Undoubtedly, several earlier studies have investigated the primary importance of microhabitats being a determinative factor for the differences in metal contents in parasites (Sures et al. 1998, 1999a). The findings of this study paralleled to those, indicating *P. nemipteri*, firmly adhering to the dorsal fins, ventral fins, and the operculum, absorbs toxic elements (Cd, Pb, Sr, Al) directly through the blood of the host which was assumed to be the main source for the nematode. The high uptake of the toxic elements (Pb, Cd, Al, As, Hg, and Sr) also by *P. nemipteri* seems to be through the surroundings water (as an additional route), similar to the findings of an experiment in study conducted by Zimmermann et al. (1999).

Contrastingly, the *H. reliquens* was discovered being attached to the host’s gastrointestinal tract absorbing all the available metals from the host intestine which is the most likely site for metal uptake by these parasites (Sures et al. 1999a), more specifically via ingestion of food as well as via body surface from the host’s intestinal lumen. As discussed earlier, high concentrations of Ni, Fe, Mn, and Zn were accumulated by *H. reliquens*; it may be attributed to the presence of bile acids increases the bioavailability of metals as suggested by Sures (Sures and Siddall 1999; Sures et al. 1999a).

The concentrations of nonessential elements like Pb, Cd, Al, and Sr in *H. reliquens* were significantly higher in comparison to the muscle and the liver and kidneys, but not higher than those in the *P. nemipteri*, which supports the assumption that some heavy metals are predominantly available for intestinal parasites. Relatively low levels of toxic elements in *H. reliquens* may be interpreted due to interspecific competition between both parasites.

As shown earlier in Table 4, strong positive correlations determined in the present study for *H. reliquens* and *P. nemipteri* indicate a competitive interaction of the parasites among essential elements. The levels of Cu, Fe, Mn, Ni, and Zn in the nematodes increase with an increasing presence in the muscles, liver, and kidneys of host; as these elements are of physiological importance for most animals (Merian 2004), it is conceivable that competition among parasites for these elements may lead to an increased absorption of other, nonessential or even toxic elements such as Pb and Cd as far as they can be detoxified (Sures 2002).

This study provides the first report regarding the bioaccumulation capacities of the most common nematodes of notched threadfin bream in the South China Sea. On the basis of the presented findings, it can be concluded that *H. reliquens*, *P. nemipteri* have high tendency to accumulate both essential and nonessential metals. Some toxic metals such as Cd, Sr, and As were accumulated up to 292, 248, and 34 times higher by *P. nemipteri* (versus host muscle), respectively, and Mn, Cu by 68, 60-fold higher in *H. reliquens* than in host muscle respectively. The divergence in heavy metal accumulation profiles of both nematodes is linked with the specificity of microhabitats, cuticle morphology, and interspecific competition. This study revealed that the presence of nematodes possibly hinder heavy metal burden in fish to a considerable extent by absorbing the elements themselves. These parasitic models can be promising future candidates for biomonitoring of metal pollution in marine ecosystems.
